# Gastric and Diaphragmatic Perforations; with Medico-Legal Remarks

**Published:** 1829-01-01

**Authors:** 


### XXXIX.

### BELFAST HOSPITAL.

### GASTRIC AND DIAPHRAGMATIC PERFORATION.

A curious case of this kind has lately been published by Dr. M'Cormac, of Belfast, and it is to be regretted that the Doctor has taken no pains to learn the symptoms or condition of the patient during life, so that the interest of the case is quite lost, except in a medico-legal point of view. It appears that a man was knocked down in a scuffle, and was carried into the Belfast Hospital; but how long after the accident, is no where stated. He laboured under symptoms of compression of the brain, for which he *was bled and had wine given to him.* He died the next morning.

On dissection, no fracture of the skull was found, although there was an external wound on one of the temples. "An almost continuous coating of coagulated blood was visible on the pia mater, covering the upper surface of both hemispheres. There were about two ounces of bloody serum at the base of the brain." On opening the abdomen, Dr. M'Cormac detected perforations in the cardiac extremity of the stomach to an unprecedented extent. The thorax being previously opened, a pint of reddish fluid was found in the right cavity, and Dr. M. traced the mode of its introduction through a hole in the diaphragm, large enough to admit his finger to pass into the stomach. This hole had a black, sphacelated, and ragged appearance round the margin. "There was erosion but *not perforation* of another portion of the diaphragm. This erosion was two inches in diameter, the *peritoneal* and *pleuritic* coats being nearly obliterated." The perforations in the stomach were two principal, and some smaller ones. "The first were about *three inches in diameter*, having irregular indented edges, with filmy prolongations of the peritoneum. The mucous membrane round the perforations was blackened and partially eroded." These perforations corresponded with the alterations of structure in the diaphragm, but there does not appear to have been any *adhesions* between the diaphragm and stomach. The soldier was not brought to trial, in consequence of the evidence given by Dr. M. before the coroner; but the Doctor says, that had a trial taken place, he would have endeavoured to prove that the blows on the head were "a secondary and inferior cause in producing death." The Doctor exhibits great learning and ingenuity in his efforts to prove, that the erosions and perforations above described took place before death, and were, in fact, the cause of the death. On these points, we differ from the Doctor. The perforations had not at all the character of ulcerations. They had no raised and hardened edges. There were too many of them. There was no adhesion between the stomach and diaphragm, which would have inevitably been the case, had there been an ulcerative process going on during life. But there is one circumstance noted, which a lawyer would have got hold of, though it seems to have escaped Dr. M. How came the pleuritic surface of the diaphragm to be eroded in one place, but *not perforated?* By the fluid, certainly, that had got in through the large perforation. This alone offers incontestible proof, that the perforations and erosions were all *post mortem.** We consider,

---

* "In a case, by Mr. Burns, the parts were found sound, on the first examination of the body, at the usual period after death; but upon a second examination, two days afterwards, this peculiar destruction of parts was found to a considerable extent."—*Abercrombie.* This, we think, puts the matter beyond dispute, respecting post-mortem perforations. The talented author from whom we quote (Dr. Abercrombie) comes to the same conclusion that we do. "Upon the whole, the conclusion, in regard to this singular affection, seems to be, that it takes place AFTER death."—*Path. Researches, p.* 57.

then, that the man above-mentioned died of concussion of the brain, together with extravasation or effusion of blood between the dura and pia mater. It is very strange that Dr. M. made no inquiry into this man's history before death. That inquiry would have been worth the whole disquisition on *ante-mortem* perforations.

